# Case of Strangulated Hernia, Not Attended by the Usual Symptoms

**Published:** 1852-08

**Authors:** Samuel W. Ritchey

**Affiliations:** Newtown, Ind.


ART. V.—*Case of Strangulated Hernia, not attended by the usual symptoms.* By SAMUEL W. RITCHEY, of Newtown, Ind.

I WAS called to visit a Mr. Philips, of our neighborhood; a slender and rather feeble man, of from 60 to 70 years of age. I learned that he had vomited and had taken some cathartic medicine, which had not operated. Not being able to visit him immediately, I sent him another dose of physic which, on visiting him in the evening, I learned had operated partially. I found him cold, stomach somewhat irritable, the pulse not very frequent, and tongue covered with a rather thick, yellowish coat; with a burning sensation at the stomach. He had been up several times, but had had but slight evacuations of the bowels. My attention was somehow called to a small tumor in the groin, which he called a "waxen kernel," but which I suspected to be a hernia. He said it had been there at times for twenty years; had never entirely disappeared, but would vary in size; on lying down, even, it would not disappear, but would get less. I doubt whether the old gentleman recollected, clearly, its history. He remarked, "that when he was out of health, it would get larger and pain him a little." It was hard and quite moveable, but not painful. I handled and compressed it, rudely as I thought desirable for any needful end, and as skillfully as I knew how, with a view to return it to the abdomen, in case it were a hernia; he made little or no complaint.

He said when he was first taken it had been somewhat painful. I was in doubt. If a hernia, it did not appear as if strangulated; although from the account given and the effort made, it might be irreducible. Another thought which struck me at the time was, that if strangulated, mortification might have taken place. In the darkness of the hour I ordered anodynes, to allay the irritability of the stomach, and cal followed by oil, to move the bowels; and left. The next morning, Dr. Cole visited the patient with me. He had not vomited through the night, but a little previous to our visit, had thrown up some chicken soup he had swallowed. Injections, in addition to the cathartics had been given, but still the bowels had moved but sparingly. He was still cool; no pain; hiccup had been almost constant all night. It might have been present previous to my former visit, but this I did not learn. The tumor was again examined, but no further light could be got. The idea of a misplaced or extra testicle was suggested, but could not be made out. No pain complained of, even on pressure. The patient declared more than once during his illness, that he had no idea the tumor had anything to do with his sickness. The symptoms remained much the same; the coldness and hiccup continued, the eyes watery, pulse intermittent and hurried. Stimulants internal and external, and antispasmodics, were administered to no purpose. He died about two days from the time I first saw him. A post mortem made the day following, revealed a strangulated inguinal hernia; the intestine mortified and nearly detached at the point of stricture. Dark spots were also observed on different portions of the peritoneum and intestines.

Was there strangulated hernia without pain or uneasiness in the tumor or abdomen, followed by mortification? or was the stricture so complete as at once to cut off sensibility in the part? or could it have been a case of rapidly sinking fever, with simultaneous mortification in the protruded intestine? In other cases of strangulated hernia which had come under our observation, the pain was urgent, and the relief obtained from faithful taxis most complete. If there had been pain or tenderness, we should have come to the conclusion positively, perhaps, that it was a case of hernia; but in the absence of the common symptoms, we could not but be in doubt. The strong probability is, that the mortification had been the result of the first day's, perhaps the first hour's, sickness; and that the case, so far as relief could have been rendered, was ended before we visited the patient; some of the prominent symptoms of mortification being present. Some might ask, why did we not resolve the doubt by cutting down upon the tumor? We answer, the doubt, only, would have been settled. The symptoms in the case did not seem to call for the knife; for who would operate in the absence of pain, with cold skin and constant hiccup?

